# Heterogeneity in age related central arterial stiffening: ascending aortic stiffness is a better predictor of carotid to femoral pulse wave velocity and brachial peripheral blood pressure than carotid stiffness

**DOI:** 10.1186/1532-429X-11-S1-P245

**Published:** 2009-01-28

**Authors:** Alban Redheuil, Wen Chung Yu, Raymond T Yan, Elie Mousseaux, Alain De Cesare, David A Bluemke, Joao AC Lima

**Affiliations:** 1 Johns Hopkins University, Baltimore, MD USA; 2 INSERM U678, Paris, France; 3 NIH, Bethesda, MD USA

**Keywords:** Pulse Pressure, Pulse Wave Velocity, Aortic Stiffness, Stiffness Index, Central Pulse Pressure

## Introduction

Increased systolic blood pressure (SBP), pulse pressure and carotid-femoral pulse wave velocity (cfPWV) are strong predictors of cardiovascular events. The aorta and carotids are both elastic arteries susceptible to arterial stiffening and its deleterious complications. The relative importance of ascending aortic and carotid stiffness on central arterial stiffness remains unclear.

## Purpose

Our aim was to study the relationship of local arterial stiffness in the carotid and ascending aorta to central arterial stiffness assessed by cfPWV and peripheral BP.

## Methods

We studied 22 healthy subjects (12 men; mean age: 37 yrs [22–67]). Central arterial stiffness was determined using carotid and femoral tonometry and transit surface distances (cfPWV). Distensibility of the ascending aorta was calculated as the ratio of its transverse area change on MRI to the central pulse pressure from radial tonometry arterial waveforms using a transfer function. Carotid distensibility was calculated as the ratio of transverse area change by sonography to carotid pulse pressure by tonometry. Local stiffness indexes of carotid and aorta were calculated from distensibility by the reverse Moens-Korteweg equation. Peripheral SBP and pulse pressure were averages of 6 brachial measurements. Linear regression was used to study correlations between aortic and carotid stiffness and cfPWV

## Results

Aortic stiffness has a significantly stronger correlation than carotid stiffness with: age (r = 0.57; p < 0.001 vs. 0.20; p = 0.02), peripheral SBP (r = 0.78; p < 0.001 vs. 0.53; p < 0.001), pulse pressure (r = 0.67 vs. 0.47; p < 0.001), and cfPWV (r = 0.87; p < 0.001 vs. 0.35; p = 0.002). In multivariate analysis with simultaneous adjustment for age, body mass index, SBP, pulse pressure, aortic stiffness and carotid stiffness, aortic stiffness is the strongest independent determinant of cfPWV (p = 0.013), SBP (p = 0.005) and pulse pressure (p = 0.01).

## Conclusion

Stiffness of the ascending aorta is a stronger predictor of cfPWV and peripheral SBP and pulse pressure than carotid stiffness among healthy individuals. This is consistent with heterogenous age-related stiffening of large arteries.

